# Gain-of-function, focal segmental glomerulosclerosis *Trpc6* mutation minimally affects susceptibility to renal injury in several mouse models

**DOI:** 10.1371/journal.pone.0272313

**Published:** 2022-08-01

**Authors:** Brittney J. Brown, Kimber L. Boekell, Brian R. Stotter, Brianna E. Talbot, Johannes S. Schlondorff

**Affiliations:** 1 Division of Nephrology, Beth Israel Deaconess Medical Center, Harvard Medical School, Boston, Massachusetts, United States of America; 2 Division of Nephrology, Boston Children’s Hospital, Harvard Medical School, Boston, Massachusetts, United States of America; University of Utah School of Medicine, UNITED STATES

## Abstract

Mutations in *TRPC6* are a cause of autosomal dominant focal segmental glomerulosclerosis in humans. Many of these mutations are known to have a gain-of-function effect on the non-specific cation channel function of TRPC6. *In vitro* studies have suggested these mutations affect several signaling pathways, but *in vivo* studies have largely compared wild-type and *Trpc6*-deficient rodents. We developed mice carrying a gain-of-function *Trpc6* mutation encoding an E896K amino acid change, corresponding to a known FSGS mutation in *TRPC6*. Homozygous mutant *Trpc6* animals have no appreciable renal pathology, and do not develop albuminuria until very advanced age. The *Trpc6*^*E896K*^ mutation does not impart susceptibility to PAN nephrosis. The animals show a slight delay in recovery from the albumin overload model. In response to chronic angiotensin II infusion, *Trpc6*^*E896K/E896K*^ mice have slightly greater albuminuria initially compared to wild-type animals, an effect that is lost at later time points, and a statistically non-significant trend toward more glomerular injury. This phenotype is nearly opposite to that of *Trpc6*-deficient animals previously described. The *Trpc6* mutation does not appreciably impact renal interstitial fibrosis in response to either angiotensin II infusion, or folate-induced kidney injury. TRPC6 protein and TRPC6-agonist induced calcium influx could not be detected in glomeruli. In sum, these findings suggest that a gain-of-function *Trpc6* mutation confers only a mild susceptibility to glomerular injury in the mouse.

## Introduction

Focal and segmental glomerulosclerosis (FSGS) is a common cause of nephrotic syndrome in adults, frequently progresses to end-stage kidney disease, and has few effective treatment options [1–3]. Studies over the last 25 years have uncovered a substantial genetic component in the pathogenesis of FSGS [4–9]. Several dozen genes are implicated in the development of autosomal recessive and dominant forms of congenital nephrotic syndrome and FSGS [10]. Among these, gain-of-function mutations in *TRPC6* are known to cause autosomal dominant FSGS in humans [11–17].

Canonical transient receptor potential 6 (TRPC6) is a member of the transient receptor potential (TRP) superfamily of cation channels [18, 19]. TRPC6 is a non-specific cation channel activated downstream of Gα_q_ coupled receptors [20], including angiotensin II receptor 1 [13], and functions as a receptor-operated calcium effector [21]. Multiple *TRPC6* mutations have been reported in cases of autosomal dominant, predominantly adult onset, FSGS [12–17, 22]. The plurality of these have a gain-of-function (GOF) phenotype, and cluster to the interface of the N-terminal ankyrin repeat domain with the C-terminal rib helix and coiled-coil [23–25], disrupting an inhibitory calcium-binding site [26]. *In vitro* studies demonstrate that overexpression of TRPC6 GOF mutants activates several signaling pathways [27, 28], and induces cytotoxicity [29–31]. The relevance of these findings to disease pathogenesis, however, remains uncertain.

An animal model genetically recapitulating TRPC6 gain-of-function disease has not been reported to date. Transgenic overexpression of either wild-type, or gain-of-function, mutant *Trpc6* in podocytes causes only modest albuminuria and mild histological changes in mice, with no clear difference between wild-type and mutant *Trpc6* animals [32]. As *Trpc6* is widely expressed, including in mesangial cells, renal tubular epithelial cells, smooth muscle cells, and fibroblasts [13, 33–35], the relative importance of TRPC6 activity in podocytes versus other cells types in the pathogenesis of FSGS is unclear. While *TRPC6* has been shown to be upregulated in several acquired proteinuric diseases [36–38], whether increased channel expression mediates similar pathologic effects as mutant TRPC6 is unknown. Studies utilizing *Trpc6* knockout animals have provided conflicting data as to a role for the wild-type channel in renal disease, with some reporting amelioration of disease [29, 35, 37, 39–41], and others increased susceptibility [42, 43].

In the present study, we characterize the renal consequences of introducing a gain-of-function E896K mutation [44], corresponding to the human TRPC6 E897K FSGS mutation [12], into the mouse *Trpc6* gene. Homozygous *Trpc6*^*E896K/E896K*^ mice have no baseline renal pathology or proteinuria until advanced age. They show no susceptibility to PAN nephrosis, only mildly delayed recovery from the albumin overload model, and transiently higher albuminuria early in an angiotensin II infusion model with an associated trend toward more glomerular sclerosis. Furthermore, the *Trpc6* mutation does not influence recovery from, nor residual interstitial fibrosis induced by, folate-induced acute kidney injury. In sum, the results suggest that gain-of-function *Trpc6* mutations induce only mild susceptibility to renal disease in the mouse.

## Material and methods

### Materials

All chemicals were purchased from Sigma Aldrich unless otherwise specified. GSK1702934A (GSK) was obtained from Focus Biomolecules and dissolved in DMSO. Fura-2 QBT was from Molecular Devices. Puromycin aminonucleoside was from MedChemExpress.

### Mice

All animal procedures were approved by the Beth Israel Deaconess Medical Center (BIDMC) Animal Care and Use Committee, and carried out in accordance with the National Institutes of Health Guide for the Care and Use of Laboratory Animals. *Trpc6*^*E896K/E896K*^ mice were generated at the BIDMC transgenic core via established protocols as described [44]. Animals were backcrossed and maintained on an FVB/NJ (Jackson Laboratory) background. Genotyping of the *Trpc6*^*E896K*^ locus was performed using a custom TaqMan SNP assay. *Trpc6*^*-/-*^ mice [45] were obtained from the Jackson Laboratory. After crossing the mice with C57BL/6J, heterozygous *Trpc6*^*+/-*^ mice were crossed to generate *Trpc6*^*-/-*^ mice and littermate *Trpc6*^*+/+*^ wild-type animals. Animals were maintained in a temperature controlled facility with a 12 hour light, 12 hour dark cycle, and had ad lib access to water and standard chow.

For tissue collection, mice were sacrificed by deep anesthesia with inhaled isoflurane on a warming blanket to minimize distress, followed by terminal cardiac puncture. Serum samples were obtained by cheek pouch vein or terminal cardiac puncture, and sent to the UAB O’Brien Center Core C for serum creatinine measurement by isotope dilution LC-MS/MS. Spot urine samples were collected from mice in unlined cages for up to 3 hours. Albuminuria quantification was performed using a mouse albumin ELISA kit (Bethyl Laboratories, Inc. E90-134). Urine creatinine measurement was performed by quantitative colorimetric assay using the QuantiChrom Creatinine Assay Kit (BioAssay Systems DICT-500).

#### Albumin overload model

8–12 week old, male wild-type (n = 11) and *Trpc6*^*E897K/E897K*^ (n = 15) mice were given daily injections of low-endotoxin bovine serum albumin (BSA, A-9430, Sigma Chemical Co, St. Louis, MO) in sterile saline (300mg/ml) by intraperitoneal injection at a dose of 10mg/g body weight on days 1–5 using an established protocol [46]. Urine samples were collected at baseline, day 2 (prior to the second injection), day 6 (24 hours after the last injection), day 9 and day 12. Urine albumin and creatinine measurements were obtained as above.

#### Puromycin aminonucleoside nephrosis

We utilized the two dose PAN model as described by Refaeli et al. [47] 8 to 10 week old male mice of various genotypes were utilized: *Trpc6*^*E896K/E896K*^ on an FVB background; and wild type and *Trpc6*^*+/E896K*^ F1 offspring of FVB and 129X1/SvJ (Jackson Laboratory) crossings. Animals were given two doses of puromycin aminonucleoside (450mg/kg; dissolved in normal saline at 18mg/ml) by intraperitoneal injection on days 0 and 7. Urine was collected at baseline, and at day 14 and 21; animals were sacrificed and kidneys collected at day 14 or 21.

#### Angiotensin II infusion model

Three month old male wild-type and *Trpc6*^*E896K/E896K*^ mice (n = 10 each) were implanted with osmotic minipumps (Alzet model 2004; Alza Corp) loaded with angiotensin II diluted in sterile saline to provide a dose of 1 μg/kg/min. The minipumps were implanted in a dorsal subcutaneous location under isofluorane anesthesia under sterile conditions. A dose of meloxicam, 1 mg/kg subcutaneously, was given prior to anesthesia to provide post-operative analgesia; a heating pad was utilized during the operative and post-operative period to prevent hypothermia. Urine was collected at baseline, and at weeks 2 and 4 of the infusion. Serum was collected at baseline and at the time of sacrifice. At the end of the 4 week infusion period, animals were sacrificed, and heart and kidneys were harvested and weighed. Tissue samples were processed for histologic analysis. Additional tissue samples were snap frozen in liquid nitrogen for RNA isolation.

#### Folate nephropathy model

Three to four month old, male and female, wild-type and *Trpc6*^*E896K/E896K*^ mice (n = 13–19 per group) were administered folate (20 mg/ml in 0.3 M sodium bicarbonate solution) at a dose of 250 mg/kg by intraperitoneal injection. Serum samples were collected at baseline (1 week prior to folate administration), 2 days after folate injection, and at the time of sacrifice. 21 days after folate injection, animals were sacrificed under anesthesia. Kidneys were decapsulated and processed for histology and RNA isolation.

### Histology

Tissue was transversely bread loafed and immersion fixed in 4% paraformaldehyde at 4°C for 24 hours. After washing in PBS, samples were further processed for paraffin embedding, sectioning, and staining by the BIDMC Histology Core. Three-micron sections stained with H&E, PAS, and Sirius Red were analyzed using an Olympus BX60 microscope equipped with a digital DP73 camera and cellSens software.

Glomeruli were scored for sclerotic lesions (present or absent) on PAS stained sections by an observer blinded to genotype and treatment. All glomeruli (>100/animal) on a single histologic section containing 2–3 transversely bread loafed portions of a kidney were scored, and the percentage of sclerosed glomeruli calculated.

To calculate podocyte density, kidney sections were stained for immunofluorescence microscopy as described previously [48], using rabbit monoclonal anti-WT1 (Abcam, ab89901) and appropriate fluorophore-labeled secondary antibody (Jackson ImmunoResearch Laboratories), and counterstained with fluorescently labeled wheat germ agglutinin (Invitrogen, W7024) and Hoechst 33342. Podocyte number and glomerular cross-section were obtained for 20 glomeruli per animal. TRPC6 immunofluorescence microscopy was performed using a rabbit polyclonal antibody (Alomone, ACC-017).

Renal fibrosis was quantified by imaging Sirius Red stained sections under polarized light using established methods [49]. Ten non-overlapping 20x fields of cortex were captured per animal, and analyzed using ImageJ. Perivascular areas were excluded from the analysis.

### Platelet and glomerular isolation

Mouse platelets were isolated using modified standard procedures [50], as previously outlined [44]. Washed platelets were resuspended directly in NP-40 lysis buffer containing Complete protease inhibitors (Roche). Glomeruli were isolated using magnetic bead perfusion as previously described [48, 51], utilizing high iron content magnetic particles (AMS-40-10H, Spherotech). For western blot, glomeruli were resuspended in NP-40 lysis buffer with protease inhibitors. For podocyte outgrowth, glomeruli were cultured in 6 well tissue culture plates with RPMI-1640 supplemented with 10% fetal bovine serum, and 1% ampicillin, penicillin, and streptomycin [52]. After 10 days of culture, outgrown podocytes were passaged and plated onto clear bottom 96 well plates for calcium imaging experiments.

### Fura-2 calcium imaging

Primary podocyte cultures were subject to Fura-2 fluorescence ratio measurement (Fura-2 QBT kit R8197, Molecular Devices) using a FlexStation III reader with automated pipetting at the Harvard ICCB-Longwood screening facility in a 96-well format essentially as previously described [30, 44]. Final concentrations of agonists were as follows: 100 μM ADP, 100 μM ATP, 50 μM GSK1702934A, 0.5 u/ml thrombin, and 1 μM thapsigargin.

### RNA isolation and gene expression

RNA was isolated from snap frozen tissue using RNeasy universal kits, and reverse transcribed into cDNA with the QuantiTect Reverse transcription kit (both Qiagen). Real-time PCR reactions were run on a QuantStudio 6 Flex machine using PowerUp SYBR Green master mix (Applied Biosystems) using gene specific primer sets (Table 1). Expression levels were normalized to 18S rRNA, *Hprt* and *Gapdh*.

**Table 1:** Primer sequences used for qPCR gene expression analysis.

Target	Forward	Reverse
GAPDH	CGTCCCGTAGACAAAATGG	TCAATGAAGGGGTCGTTGA
HPRT	GAGGAGTCCTGTTGATGTTGCCAG	GGCTGGCCTATAGGCTCATAGTGC
αSMA	CTGACAGAGGCACCACTGAA	CATCTCCAGAGTCCAGCACA
Collagen I	GAGCGGAGAGTACTGGATCG	GTTCGGGCTGATGTACCAGT
Fn1	CGAGGTGACAGAGACCACAA	CTGGAGTCAAGCCAGACACA
TGF-β	TTGCTTCAGCTCCACAGAGA	TGGTTGTAGAGGGCAAGGAC
CTGF	CCCTAGCTGCCTACCGACTG	TTAGAACAGGCGCTCCACTC
KIM-1	TCAGCTCGGGAATGCACAA	TGGTTGCCTTCCGTGTCTCT
NGAL (Lcn2)	TGATCCCTGCCCCATCTCT	GGAACTGATCGCTCCGGAA
18S RNA	GCAATTATTCCCCATGAACG	AGGGCCTCACTAAACCATCC
Trpc6	ACTGGTGTGCTCCTTGCAG	GAGCAGCCCCAGGAAAAT

### Western blotting

Lysates were mixed with 4x sample loading buffer containing β-mercaptoethanol and immediately incubated at 95°C for 5 minutes. SDS-PAGE was performed as previously described [28]. Western blotting using fluorescence detection was performed using Immobilon-FL PVDF membrane (Millipore), Chameleon Duo pre-stained protein ladder, Intercept blocking buffer, and an Odyssey CLx imaging system (all LI-COR). Primary antibodies against the following antigens were utilized: Erk1/2 (CST #9107, 1:1000), Podocalyxin (MAB1556, R&D Systems, 1:500), TRPC6 (Alomone ACC-017, 1:500). Fluorescent secondary antibodies (IRDye 690RD anti-mouse and anti-rat; IRDye 800CW anti-rabbit; all LI-COR) were used at 1:20,000 dilution.

### Statistical analysis

All statistical analyses were performed using GraphPad Prism version 9. The specific statistical tests utilized for each experiment are specified within the corresponding figure legends. Symbols used for pair-wise comparison adjusted p-values are: ns, p>0.05; *, p<0.05; **, p<0.01; ***, p<0.001.

## Results

**Fig 1:**
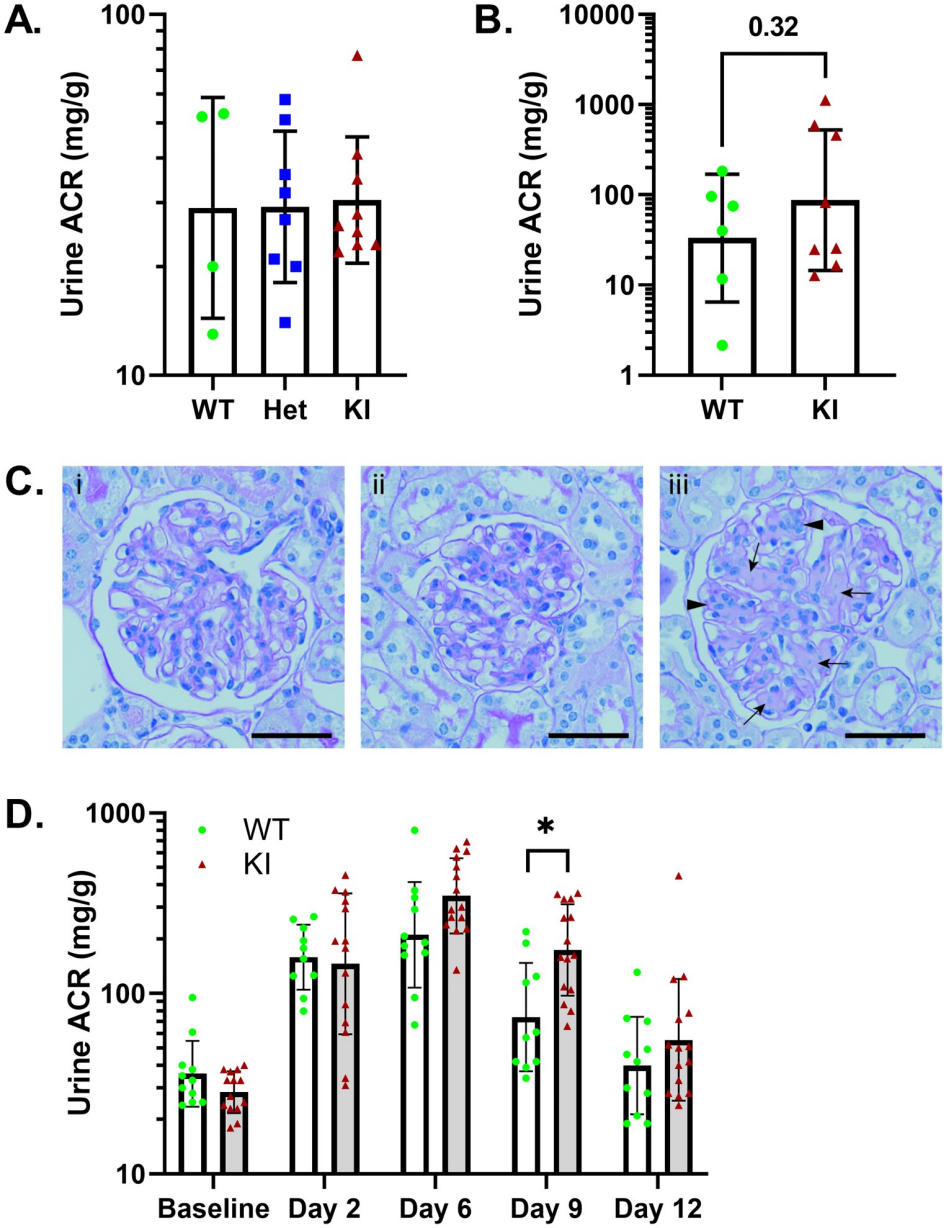
Baseline and albumin overload induced albuminuria in wild-type and *Trpc6*^*E896K/E896K*^ mice. A, urine albumin-to-creatinine ratio (ACR) measurements from six-month old, wild-type (WT), *Trpc6*^*+/E896K*^ (Het), and *Trpc6*^*E896K/E896K*^ (KI) males. Shown are individual values, geometric mean and SD; n = 4-9/group. Groups were compared by one-way ANOVA with Tukey’s multiple comparisons test; all comparisons without statistical significant differences. B, urine ACR from 20–23 month old, female WT (n = 6) and KI (n = 8) animals. Shown are geometric mean and individual values; log-transformed ACRs were compared by unpaired t-test. C, glomerular histology of 23 month old, female (i) wild-type, (ii) non-proteinuric KI, and (iii) albuminuric KI mice. Mesangial expansion (arrows) and mesangial hypercellularity (arrowheads) are apparent in the albuminuric animal. PAS stained sections; scale bar represents 20 μm. D, albuminuria in WT and KI male mice subject to albumin overload from day 1–5. Shown are geometric mean, SD and individual values; n = 11 (WT) and 15 (KI). Log-transformed ACRs were compared between genotypes by mixed-effects analysis with Sidak’s multiple comparisons test. *Trpc6*^*E896K/E896K*^ had statistically significantly more albuminuria compared to wild-type only on day 9.

*Trpc6*^*E896K/E896K*^ mice were viable and fertile, and born at the expected Mendelian ratio when generated by mating heterozygous animals. *Trpc6*^*+/E896K*^ and *Trpc6*^*E896K/E896K*^ mice showed no evidence of developing albuminuria compared to their wild-type counterparts at 6 months of age (Fig 1A). Even in female animals aged 20 to 23 months, albuminuria did not differ significantly between wild-type and knock-in animals (Fig 1B). The fraction of *Trpc6*^*E896K/E896K*^ mice that did develop albuminuria demonstrated mesangial expansion and mesangial hypercellularity, but no appreciable glomerular sclerosis (Fig 1C), similar to age-associated glomerular changes reported to occur in wild-type female mice in this age range [53, 54].

### Albumin overload model

Wild-type and *Trpc6*^*E896K/E896K*^ male mice were compared in their response to the transient albumin overload model (Fig 1D). A mixed-effects model suggests a statistically significant effect of genotype on albuminuria across the combined time-points (p = 0.0153). However, by multiple comparisons, the albuminuria observed in *Trpc6*^*E896K/E896K*^ male mice was only statistically significantly higher than controls at day 9, 4 days after the last albumin administration. Both groups showed a similar degree of albuminuria a week after injections were discontinued, when albuminuria had returned back to near baseline. As has been reported by others [55], no histological sequelae were apparent by light microscopy after the recovery period.

### Puromycin aminonucleoside nephrosis

**Fig 2:**
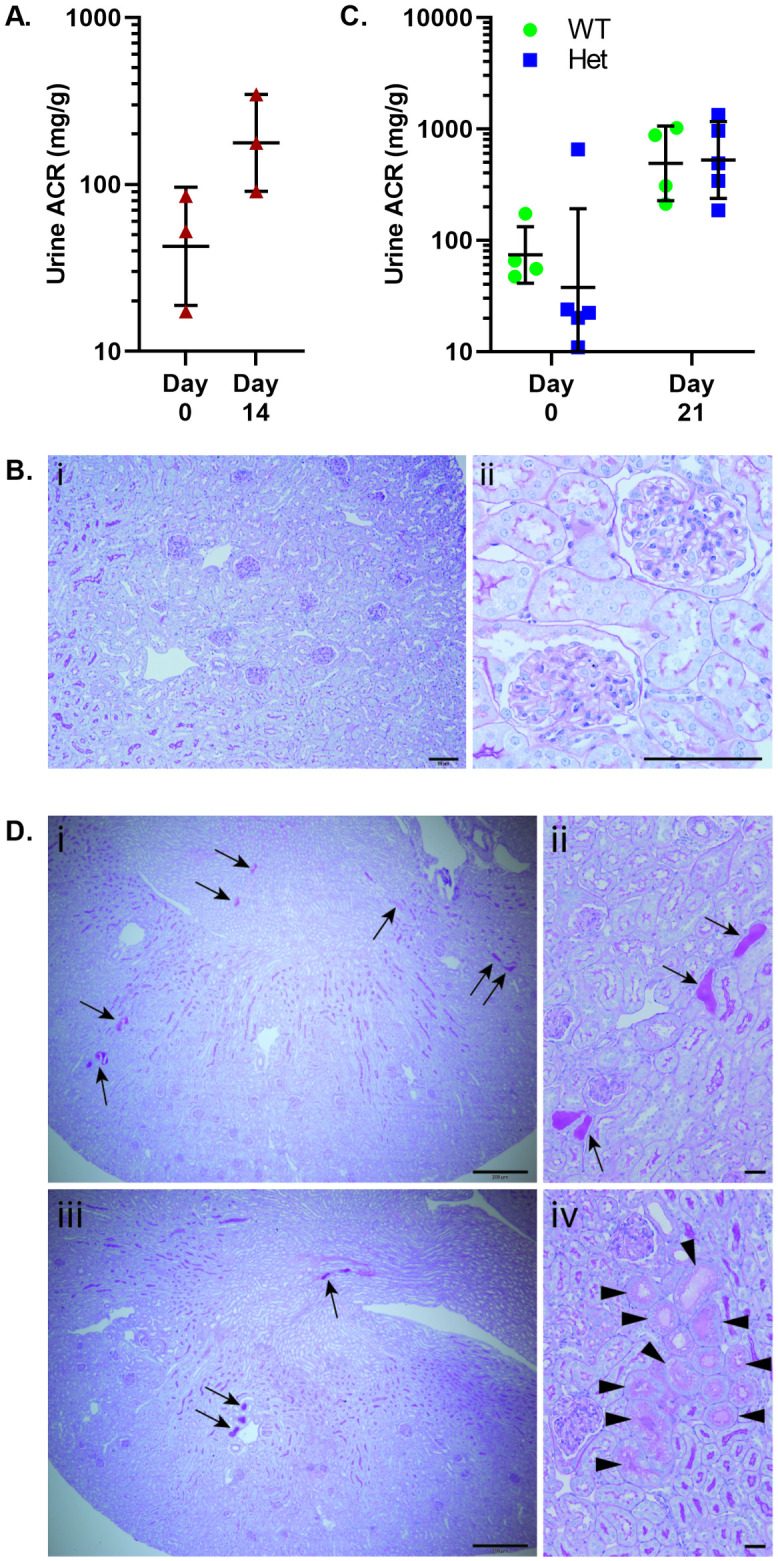
Puromycin aminonucleoside nephrosis in *Trpc6*^*E896K*^ mutant mice. Various *Trpc6* genotype male mice were subject to two intraperitoneal injections of puromycin aminonucleoside (PA; 450mg/kg) on days 0 and 7. *Trpc6*^*E896K/E896K*^ mice on the FVB background developed a small (<5 fold) increase in albuminuria at day 14 compared to baseline (A). B, PAS stained histology sections of *Trpc6*^*E896K/E896K*^ kidney 14 days after PAN revealed no appreciable (i) cortical, or (ii) glomerular, pathology. Scale bar represents 50 μm. C, urinary albumin-to-creatinine ratios (ACR) of *Trpc6*^*+/+*^ (WT) and *Trpc6*^*+/E896K*^ (Het) animals on an F1 FVB/NJ x Sv/129 background before and 21 days after PAN induction. D, PAS stained WT (i, ii), and Het (iii, iv) kidney sections 21 days after PA administration demonstrate largely preserved cortical, and glomerular architecture, with very rare (estimated <1% of renal cortex cross sectional area) proteinaceous casts (arrows), and tubules with protein reabsorption droplets (arrowheads). Scale bars represent 200 μm (i, iii), and 20 μm (ii, iv). ACRs (A, C) are shown as geometric mean, SD and individual values. Log-transformed ACRs were compared for statistical analyses by paired t-test or two-way ANOVA.

Mice heterozygous for the *Inf2*^*R218Q*^ mutation [56], or a *Podxl* null mutation [47], show dramatic sensitivity to glomerular injury in the PAN model. We therefore examined whether the *Trpc6*^*E896K*^ allele might confer a similar susceptibility. *Trpc6*^*E896K/E896K*^ mice exposed to the two dose PAN regimen [47] had only a small increase in proteinuria (Fig 2A) without the development of glomerular sclerosis (Fig 2B). We also compared *Trpc6*^*+/+*^ and *Trpc6*^*+/E896K*^ animals generated through an F1 cross with Sv/129 animals, as Sv/129 animals show some susceptibility to PAN. These animals all developed low grade proteinuria (Fig 2C), but histological analysis revealed no glomerular sclerosis, and only very rare evidence of tubular protein reabsorption droplets and proteinaceous casts (Fig 2D). Furthermore, albuminuria did not differ based on *Trpc6* genotype. These results suggest that the *Trpc6*^*E896K*^ allele does not affect susceptibility to PAN.

### Angiotensin II infusion

**Fig 3:**
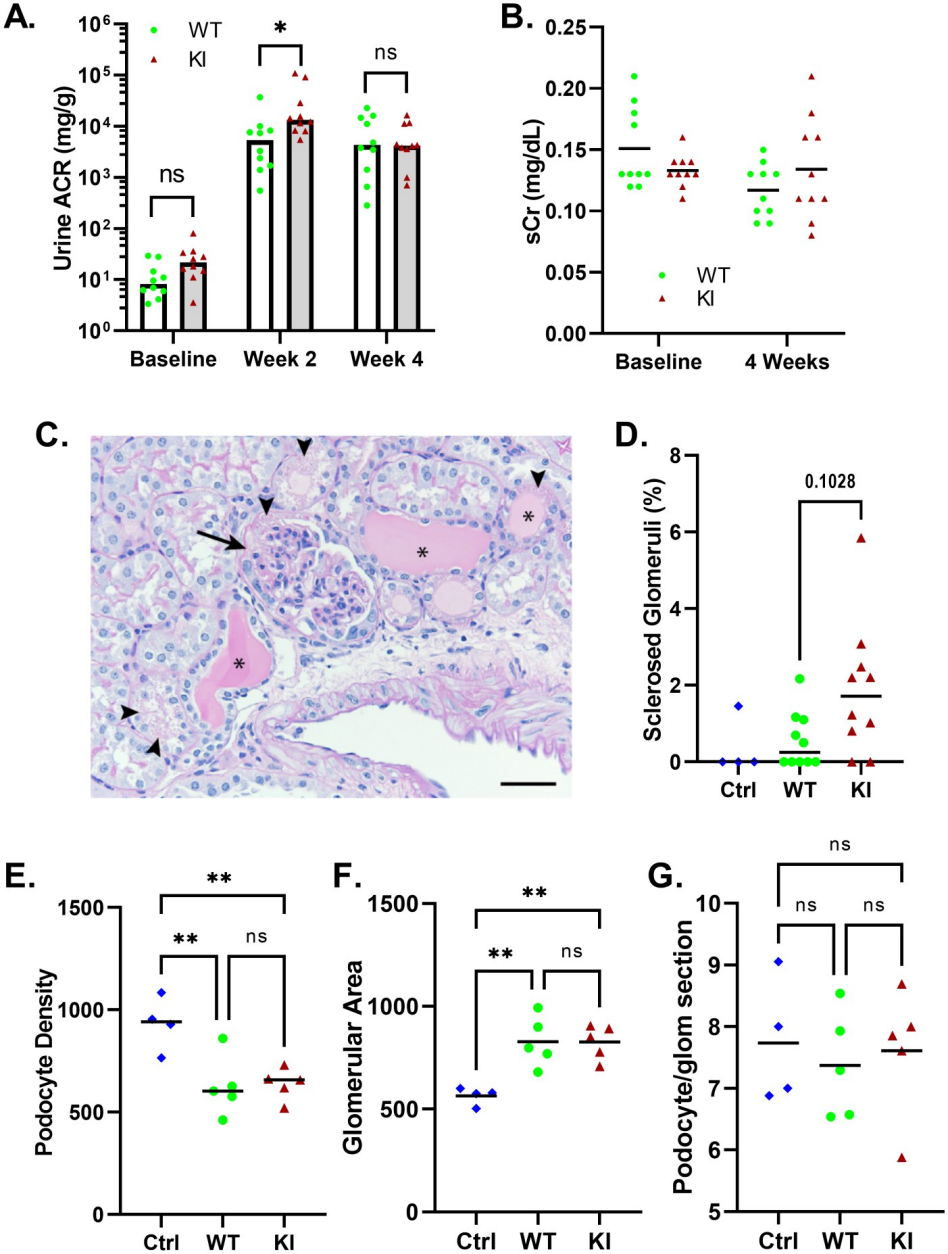
Effect of *Trpc6* genotype on glomerular response to angiotensin II infusion. Wild-type and *Trpc6*^*E896K/E896K*^ (KI) male mice were subject to ATII infusion for 4 weeks. A, urine albumin-to-creatinine ratio (ACR) measurements demonstrate development of robust albuminuria in both groups. KI mice developed slightly greater albuminuria at 2 weeks compared to wild-type, an effect that did not persist at 4 weeks. Shown are median and individual values; n = 10/group. Log-transformed ACRs were compared between genotypes by two-way ANOVA with Sidak’s multiple comparisons test. B, serum creatinine measurements at baseline and after 4 weeks of ATII infusion. Shown are mean and individual values; n = 10/group; no statistically significant differences between genotypes. C, example of renal pathology in a KI mouse after ATII infusion. Shown is a PAS stained section demonstrating a segmental glomerular lesion (arrow), tubular atrophy with cystic changes and proteinaceous casts (asterisks), and protein reabsorption droplets (arrowheads). Scale bar represents 20 μm. D, percentage of glomeruli showing evidence of segmental lesions in control wild-type males (Ctrl; n = 4), and wild-type (WT) and *Trpc6*^*E896K/E896K*^ (KI) males subjected to ATII infusion (n = 10 each). Shown are median and individual values; differences between groups did not reach statistical significance by Kruskal-Wallis test with Dunn’s multiple comparisons. Glomerular podocyte density (E), glomerular area (F), and podocyte number per glomerular cross-section (G) were measured. Shown are mean and individual averages per animal in untreated control animals (Ctrl; n = 4) and ATII treated wild-type and knock-in animals (n = 5 each). 20 glomeruli were measured per animal. One-way ANOVA analysis with Tukey’s multiple comparisons test.

Multiple studies have reported a role for TRPC6 channel activation downstream of angiotensin II signaling [13, 57–61]. *Trpc6* knockout mice develop less albuminuria initially, and trend toward less renal pathology, compared to wild-type animals, in response to chronic ATII infusion despite a similar response in blood pressure [39]. We therefore exposed *Trpc6*^*E896K/E896K*^ and wild-type male mice to 4 weeks of ATII infusion, and compared their response. *Trpc6*^*E896K/E896K*^ animals developed greater albuminuria after 2 weeks compared to wild-type animals, but this difference did not persist at the end of the infusion period (Fig 3A). Serum creatinine did not differ between genotypes either at baseline, or at the end of the experiment (Fig 3B).

Histologic examination of kidney sections revealed rare glomerular lesions and isolated tubular dilations with proteinacious casts (Fig 3C). Although there was a trend toward more glomerular lesions in *Trpc6*^*E896K/E896K*^ mice, this did not reach statistical significance (Fig 3D). Podocyte density was lower in angiotensin II treated animals compared to controls (Fig 3E), but *Trpc6* genotype did not affect this parameter. The effect was driven by an increase in average glomerular cross-sectional area (Fig 3F), with no evidence of significant podocyte loss (Fig 3G).

**Fig 4:**
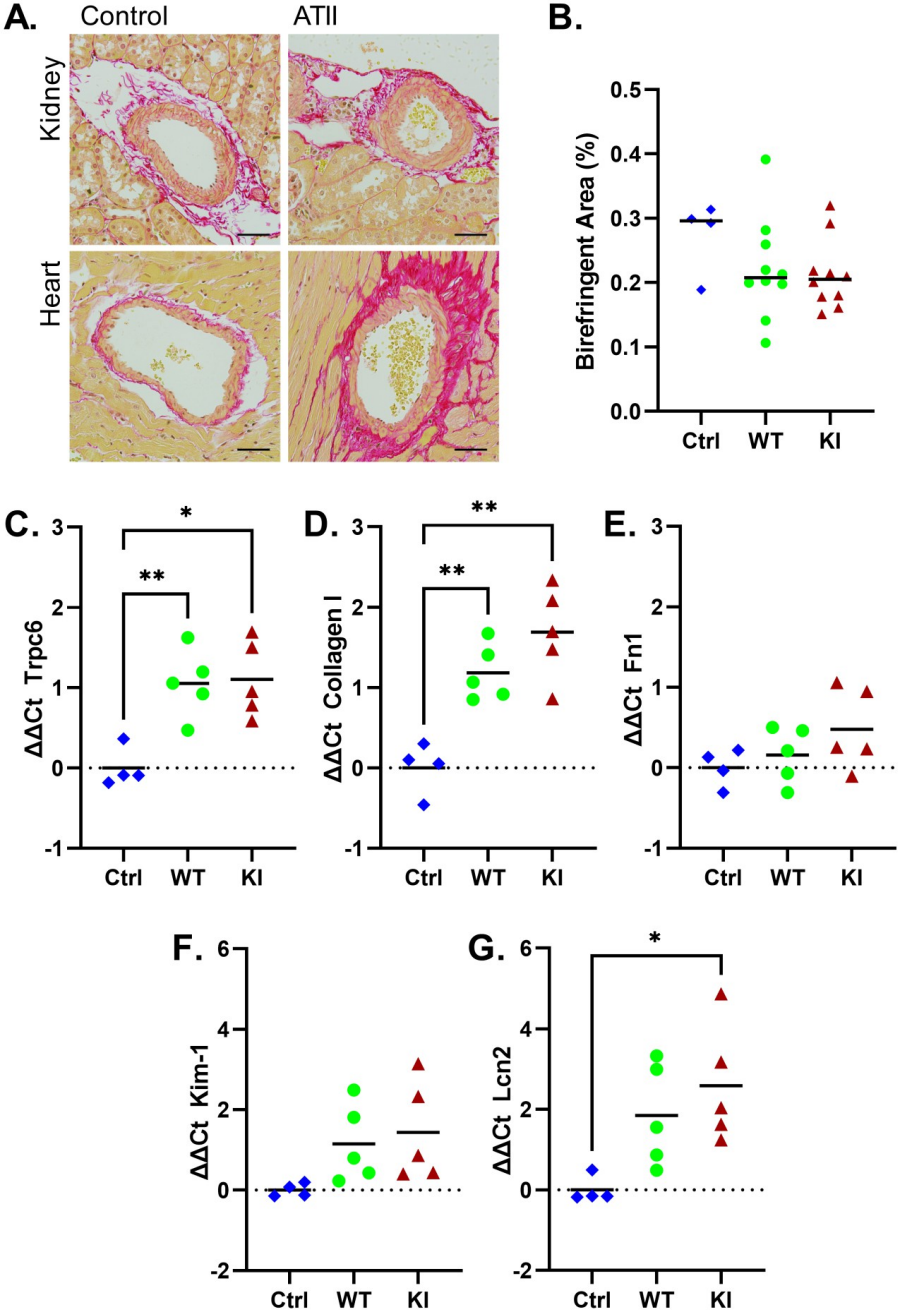
Fibrotic response to angiotensin II infusion. A, histology of control and ATII treated kidneys and heart demonstrating the development of perivascular fibrosis. Sections stained with Sirius Red; scale bar equals 20 μm. B, percentage of Sirius Red stained renal cortex demonstrating birefringence in control wild-type male animals (Ctrl), and wild-type and *Trpc6*^*E896K/E896K*^ (KI) males subjected to ATII infusion. Shown are median and individual values; no significant pairwise comparisons by one-way ANOVA with Tukey’s multiple comparisons. Relative gene expression analysis demonstrated upregulation of both *Trpc6* (C) and Collagen I (D) mRNA in kidneys subjected to ATII infusion. Fibronectin (E) showed no significant change, while Kim-1 (F) and NGAL/Lcn2 (G) demonstrated a trend toward increased expression upon ATII treatment. Shown are mean and individual values; Brown-Forsythe one-way ANOVA with Dunnett’s multiple comparisons. Only statistically significant pairwise comparisons are shown.

Angiotensin II treated animals developed significant perivascular fibrosis, especially in the heart (Fig 4A). However, interstitial fibrosis of the renal cortex, quantified by birefringence of Sirius red stained sections imaged under polarized light, showed no difference between control and ATII treated animals (Fig 4B). Kidney and heart weight, normalized to body weight, similarly were not different between groups (S1 Fig). The relative expression of *Trpc6* (Fig 4C), and several fibrosis (Fig 4D and 4E) and kidney injury (Fig 4F and 4G) genes, was ascertained by RT-PCR of whole kidney RNA. The expression of *Trpc6* and collagen I was higher in angiotensin II exposed kidneys compared to wild-type control samples, with *Kim-1* and *NGAL* (*Lcn2*) mRNA levels trending higher. However, the expression levels of none of these genes differed significantly between angiotensin treated wild-type and *Trpc6*^*E896K/E896K*^ mice.

### Folate nephropathy

**Fig 5:**
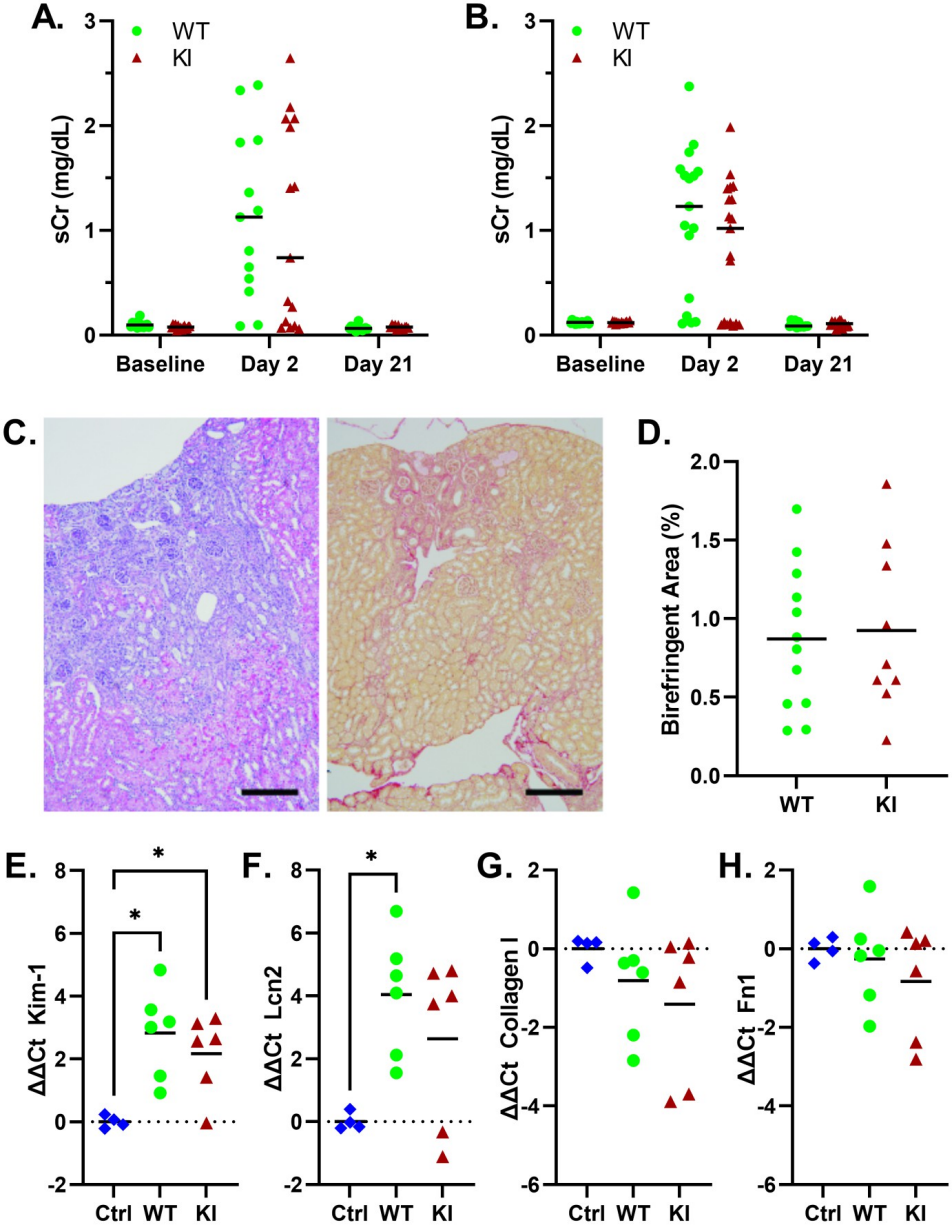
*Trpc6* genotype does not influence response to folate nephropathy. Serum creatinine in WT and KI, male (A) and female (B), mice was elevated 2 days after folate administration, and recovered to baseline levels after 3 weeks. Shown are median and individual values; n = 13-19/group. There were no statistically significant differences between genotypes at any of the time-points; two-way ANOVA with Sidak’s multiple comparisons test. C, renal histology at day 21, demonstrating areas of tubular atrophy and interstitial fibrosis surrounded by relatively preserved cortical architecture. Shown are H&E stain of a male wild-type kidney section (left), and Sirius Red stain of a female KI kidney section (right); scale bar equals 100 μm. D, the percentage of Sirius Red stained renal cortex area demonstrating birefringence 21 days after folate-induced AKI was compared between wild-type (n = 12) and KI (n = 9) male mice. Shown are mean and individual values; no differences between groups by unpaired t-test. The relative mRNA expression levels of renal injury related genes, Kim-1 (E) and Ngal/Lcn2 (F), and fibrosis genes, collagen I (G) and fibronectin (H), were compared between male control (Ctrl), and WT and KI folate-nephropathy kidney samples (n = 4-6/group). Shown are mean and individual values; Brown-Forsythe one-way ANOVA with Dunnett’s multiple comparisons. Only statistically significant pairwise comparisons are shown.

As TRPC6 has also been implicated in modulating fibrosis [35, 62–66], we compared *Trpc6*^*E896K/E896K*^ and wild-type mice in the folate nephropathy model (Fig 5). The administration of a high dose of folic acid leads to tubular injury and AKI in mice [67–69]. Although renal excretory function largely recovers, residual interstitial fibrosis and other hallmarks of chronic injury remain. Serum creatinine in both male (Fig 5A) and female (Fig 5B) mice demonstrated a robust rise two days after folate administration, returning to baseline after 3 weeks. Serum Cr did not differ significantly between *Trpc6* genotypes at any of the time points. Histologic analysis revealed foci of interstitial fibrosis and tubular atrophy (Fig 5C). The percentage of cortex demonstrating fibrosis showed significant variability within each group, and no significant difference between *Trpc6* genotypes (Fig 5D). Similarly, gene expression analysis of several fibrosis and kidney injury marker genes did not reveal any significant differences between male wild-type and *Trpc6*^*E897K/E897K*^ kidneys after folate treatment (Fig 5E–5H and S2 Fig). Tubular injury markers did show upregulation in the folate treated animals compared to controls (Fig 5E and 5F). The results do suggest significant inter-individual variability in the degree of renal scarring in this model.

### Glomeruluar TRPC6 expression and function

**Fig 6:**
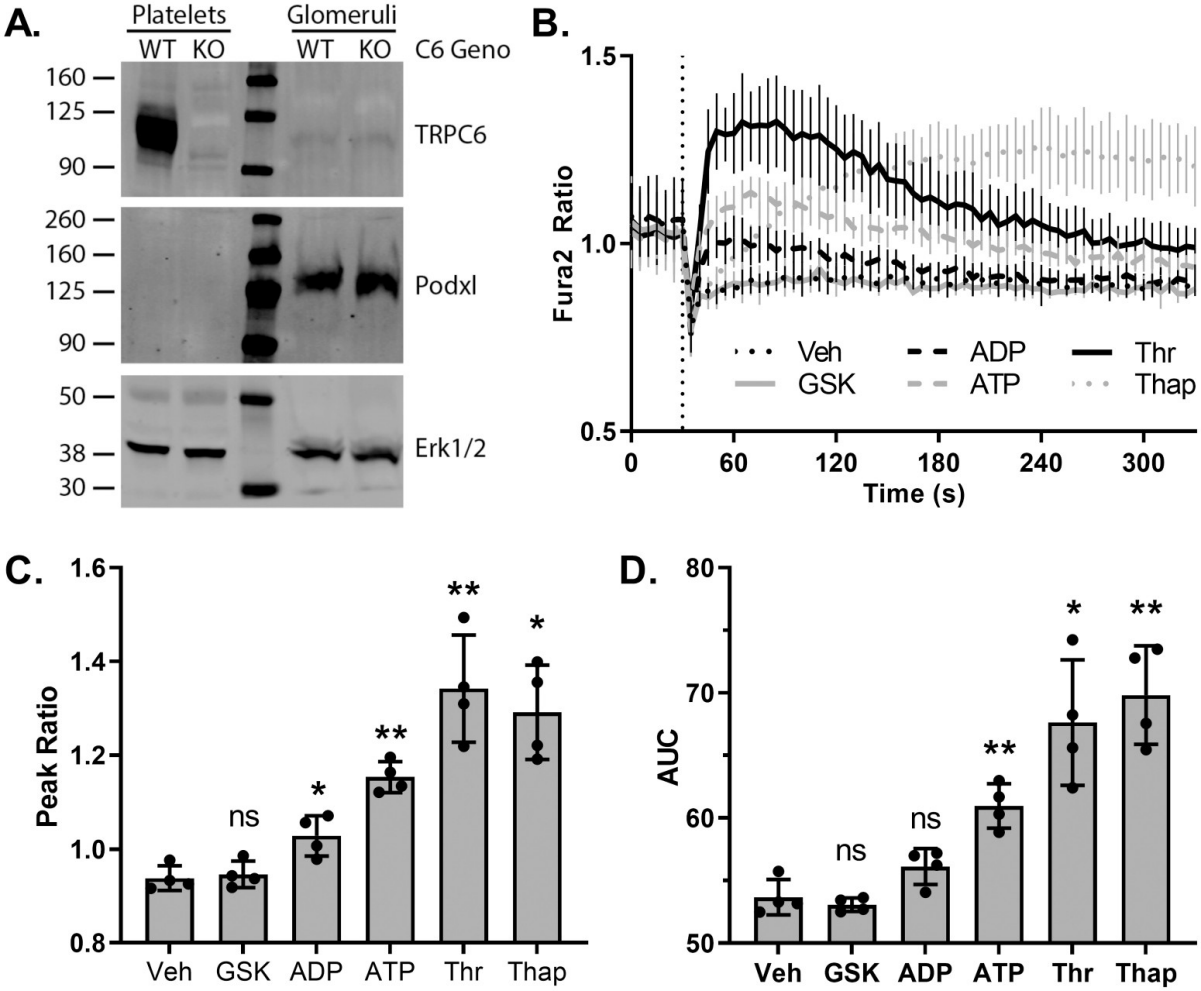
Characterization of TRPC6 expression and GSK1702934A response in murine podocytes. A, platelet and glomerular lysates from wild-type (WT) and *Trpc6*^*-/-*^ (KO) female animals were analyzed by SDS-PAGE and Western blot. Anti-TRPC6 antibodies (top) detected a major band around 105 kD in WT, but not KO, platelets. No corresponding specific band is seen in glomerular lysates. A faint band of a size similar to TRPC6 is seen in both WT and KO glomerular samples, suggesting a non-specific signal. Podocalyxin (Podxl, middle) and Erk1/2 (bottom) blots are shown as loading controls. B, time-course of Fura-2 fluorescence ratio (340/380) in primary podocytes cultured from *Trpc6*^*E896K/E896K*^ (KI) glomeruli. After 30 seconds (dashed vertical line), cells were stimulated with vehicle (Veh), GSK1702934A (GSK; 50 μM), ADP (100 μM), ATP (100 μM), thrombin (Thr; 0.5 u/ml), or thapsigargin (Thap; 1 μM). Shown are mean ± SD; n = 4 using podocytes from 2 different animals. Post-stimulation (C) peak Fura-2 fluorescence ratio, and (D) area under the curve (AUC, arbitrary units) were calculated. Shown are the mean and values from individual experiments. RM one-way ANOVA with Dunnett’s multiple comparisons test to vehicle control.

Although early reports localized human TRPC6 to the glomerular podocyte [12, 13], *in situ* hybridization [33, 35], and single cell RNAseq [70], have not detected substantial *Trpc6* mRNA in murine podocytes. We probed murine glomerular lysates for TRPC6 protein by western blot (Fig 6A), utilizing *Trpc6*^*-/-*^ mouse samples as negative controls. Although TRPC6 is readily detectable in murine platelets, glomerular extracts show no specific signal. Immunofluorescence microscopy attempts also failed to demonstrate a specific TRPC6 signal in wild-type kidney compared to *Trpc6*^*-/-*^ tissue (S3 Fig). To ascertain if TRPC6-dependent calcium influxes might be detectable despite the lack of TRPC6 signal by western blot, we performed Fura-2 fluorimetry on primary podocytes isolated from *Trpc6*^*E896K/E896K*^ glomeruli (Fig 6B–6D). We utilized GSK1702934A (GSK), a TRPC3/6 agonist [71, 72], as it induces calcium influx in mouse platelets in a TRPC6-dependent manner, with *Trpc6*^*E896K/E896K*^ platelets demonstrating a significantly larger response compared to wild-type platelets [44]. GSK failed to induce any significant change in Fura-2 fluorescence compared to vehicle control. ADP, ATP, and thrombin, utilized as positive controls, all induced Fura-2 responses in these cells, consistent with prior reports [73–77]. In sum, we have been unable to demonstrate the presence of TRPC6 protein, by western blot, immunofluorescence microscopy, or GSK-induced calcium influx in murine glomeruli. We cannot exclude the possibility of low levels of TRPC6 channel, which are not responsive to GSK, being present.

## Discussion

*TRPC6* mutations are a cause of autosomal dominant FSGS in humans [12, 13]. The plurality of these mutations lead to a gain-of-function phenotype at the level of the channel [12–17, 22]. In the current study, we report on the renal phenotype of mice carrying a mutation encoding for a gain-of-function alteration in the TRPC6 protein. Even in the homozygous state and at advanced age, these animals show minimal renal pathology relative to their wild-type counterparts, and no segmental glomerular sclerosis. Furthermore, compared to wild-type animals, they display only mild or no significant differences in their response to several renal stress models. Specifically, they demonstrate slightly delayed resolution of albuminuria in the albumin overload model, only transiently higher albuminuria and a trend toward more glomerular lesions in response to angiotensin II infusion, no enhanced susceptibility to PAN, and no significantly different response to folate-induced AKI and subsequent fibrosis. In sum, these findings suggest that the *Trpc6*^*E896K/E896K*^ mutant mouse does not readily phenocopy the renal pathology associated with gain-of-function *TRPC6* mutations in humans.

Angiotensin II signaling through AT1 receptor is thought to be a central driver in the development of proteinuria and renal injury [78]. *In vitro*, TRPC6 has been shown to be activated downstream of Gα_q_-coupled receptors [20], and AT1 receptor in particular [13, 57–61]. In a prior study, *Trpc6*^*-/-*^ mice demonstrate slightly lower albuminuria compared to wild-type animals after 2 weeks of ATII infusion, a difference that is no longer significant after 4 weeks; and a non-statistically significant trend toward less glomerular injury [39]. The results presented here, comparing wild-type and *Trpc6*^*E896K/E896K*^ murine responses to ATII infusion, neatly complement these previous results in *Trpc6* knockout animals. *Trpc6*^*E896K/E896K*^ mice show an increase in proteinuria compared to wild-type animals 2 weeks, but not 4 weeks, after the initiation of ATII treatment. Furthermore, there was a trend toward more glomerular lesions in the mutant mice, though the difference did not meet statistical significance. We did not perform blood pressure studies, and so are unable to address whether, as the earlier study [39] did, there was no effect of *Trpc6* genotype on the development of hypertension. Taken together, these results suggest that TRPC6 channel activity contributes, at least transiently, to the development of proteinuria and glomerular lesions in response to high levels of angiotensin II.

TRPC6 has been implicated in the development of fibrosis in several organs [35, 62–66, 79]. In the kidney, both genetic [35, 64], and pharmacologic [63], inhibition of TRPC6 is reported to dampen the fibrotic response in the unilateral ureteral obstruction model. We were therefore somewhat surprised that the *Trpc6*^*E896K/E896K*^ mice do not show an increased fibrotic reaction after folate-induced AKI. It is possible that mechanistically, the effects of the gain-of-function *Trpc6* mutation on kidney function are not simply the opposite of deleting *Trpc6*. This has been our experience in platelets [44]. Alternatively, TRPC6-dependent fibrosis pathways may be activated specifically downstream of ureteral obstruction, and not involved in the models tested here. Consistent with this possibility, *Trpc6* knockout in rats does not alter the age-related development of tubulointerstitial fibrosis [80]. Future studies in other fibrosis models will be needed to address these questions.

Genetically recapitulating autosomal dominant forms of FSGS in mice has frequently failed to induce glomerular pathology. In addition to the *Trpc6*^*E896K*^ mutation described here, neither the *Actn4*^*K256E*^ [81, 82], nor the *Inf2*^*R218Q*^ [52], mutations induce a renal phenotype when present in heterozygous form in mice. Homozygous *Actn4*^*K255E/K255E*^ animals, though, do develop severe glomerular pathology [81]. And both heterozygous and homozygous *Inf2* mutant mice show enhanced sensitivity to injury in several injury models [52, 56]. In the case of *Trpc6*, the homozygous knock-in animals show only a transient, and slightly, higher degree of albuminuria in the albumin overload and angiotensin II infusion models. The *Trpc6*^*E896K*^ mutation, unlike the *Inf2*^*R218Q*^ mutation [56], does not induce susceptibility to PAN. Of note, transgenic overexpression of *Trpc6* mutants, including E896K, in podocytes induces only minimal albuminuria in mice, which is not significantly different from that seen upon overexpression of wild-type *Trpc6* [32]. The reason for the relative lack of a renal phenotype in the *Trpc6*^*E896K/E896K*^ animals remains unclear. The E896K mutation is a gain-of-function mutant, as we have separately demonstrated in platelets [44]. There is a report of differences in *Trpc6* mRNA expression between mouse strains [33], and strain-specific susceptibility to kidney, and glomerular, injury are well known [55, 83–85]. It is certainly plausible that a different genetic background, or environmental insult, is necessary to elicit the pathologic effects of *Trpc6* mutations. Alternatively, it is possible that glomerular or renal TRPC6 expression differs between humans and mice, and accounts for our findings. Review of single cell RNA expression data speaks to this possibility [70, 86, 87]. And although we did not examine TRPC6 expression in human samples, we were unable to detect the protein in murine glomeruli, or detect a GSK-inducible, Fura-2 measurable, calcium influx in murine glomerular outgrowths. Future studies comparing glomerular TRPC6 expression in different mammalian species could prove informative.

In summary, introducing a gain-of-function mutation, corresponding to a human FSGS disease mutation, into murine *Trpc6* fails to recapitulate the human glomerular disease pathology. Homozygous *Trpc6*^*E896K/E896K*^ mice do demonstrate transiently, and mildly, higher albuminuria in the albumin overload and angiotensin II infusion models, but do not display a predilection for PAN, or increased interstitial fibrosis after recovery from folate-induced AKI. It remains unclear if *Trpc6* mutations require an as yet unidentified environmental or genetic hit to induce glomerular disease in mice, or if mice are intrinsically not suitable to model TRPC6-mediated human FSGS.

## Supporting information

S1 Fig *Trpc6* genotype does not influence kidney and heart weight after angiotensin II treatment. Kidney (A) and heart (B) weights, normalized to total body weight, did not differ between control wild-type male animals (Ctrl), and wild-type and *Trpc6*^*E896K/E896K*^ (KI) males subjected to ATII infusion for 4 weeks. Shown are median and individual values (n = 4-10/group); no pairwise comparison showed a statistically significant difference by one-way ANOVA with Tukey’s multiple comparisons test.
(TIF)

S2 Fig Gene expression in folate-nephropathy kidneys. The relative mRNA expression levels of several fibrosis and renal injury related genes, and *Trpc6*, was compared in WT and KI male folate-nephropathy kidney samples (n = 6/group). There were no statistically significant differences between genotypes for any of the genes; multiple unpaired t-tests.
(TIF)

S3 Fig Immunofluorescence microscopy of wild-type and *Trpc6*^*-/-*^ kidneys. A, kidney sections from wild-type (i, iii), and *Trpc6*^*-/-*^ (ii, iv) mice stained with rabbit anti-TRPC6 antibody (ACC-017, Alomone). Wheat germ agglutinin (WGA), and Hoechst were used as counterstains. TRPC6 staining specific to the wild-type kidney could not be identified. B, wild-type kidney sections were stained with TRPC6 antibody (i, iii) or with anti-rabbit secondary antibody only (ii, iv). Green channel signal was not due to non-specific secondary antibody staining or tissue auto-fluorescence.
(TIF)

S1 Raw images (PDF)
