# Supplementary figures and images for: Gain-of-function, focal segmental glomerulosclerosis Trpc6 mutation minimally affects susceptibility to renal injury in several mouse models
Source: PLoS One. 2022 Aug 1;17(8):e0272313. doi: 10.1371/journal.pone.0272313 (PMC9342776; doi:10.1371/journal.pone.0272313)

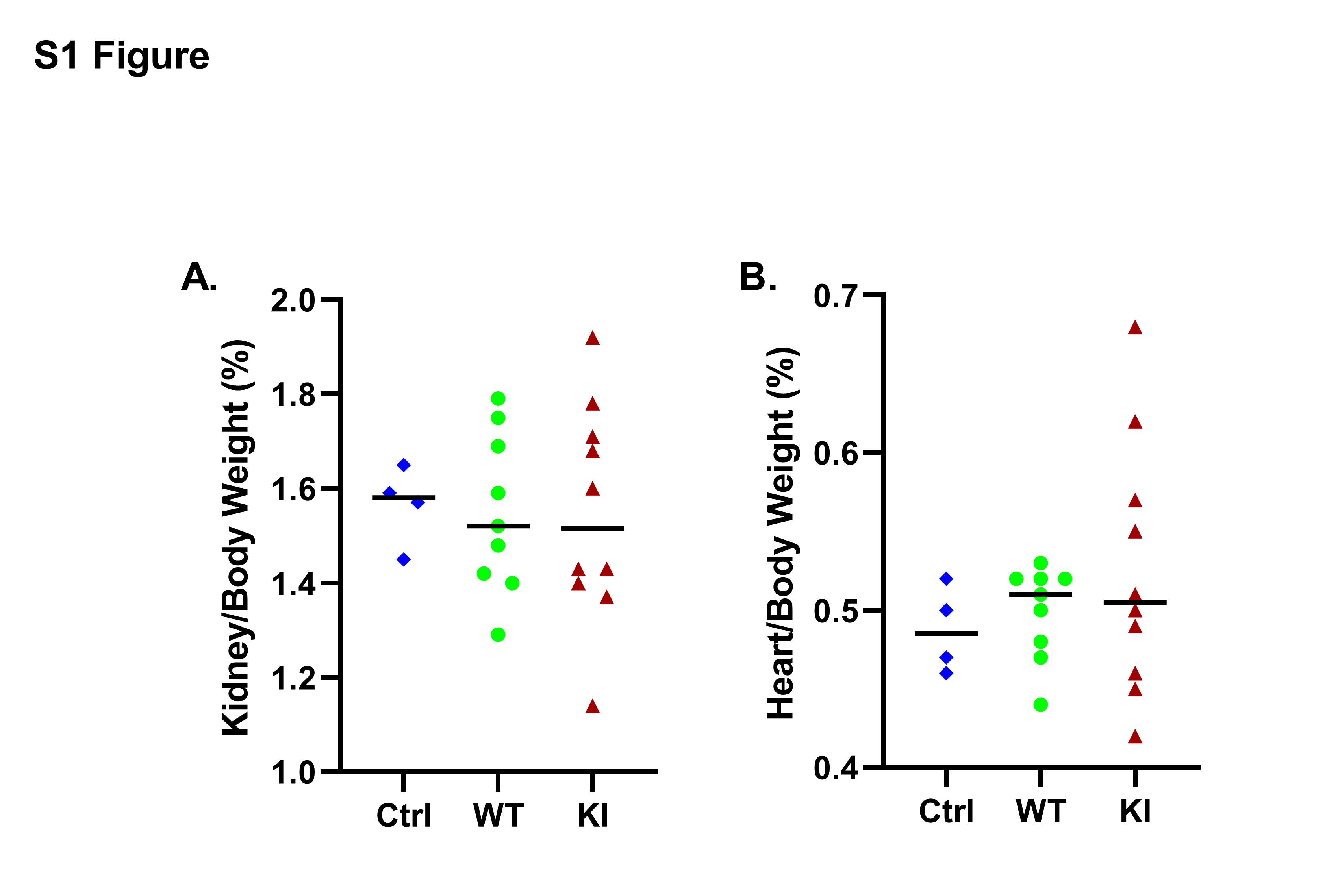

Supplement: S1 Fig — Kidney (A) and heart (B) weights, normalized to total body weight, did not differ between control wild-type male animals (Ctrl), and wild-type and Trpc6E896K/E896K (KI) males subjected to ATII infusion for 4 weeks. Shown are median and individual values (n = 4-10/group); no pairwise comparison showed a statistically significant difference by one-way ANOVA with Tukey’s multiple comparisons test. (TIF) [file pone.0272313.s001.tif]

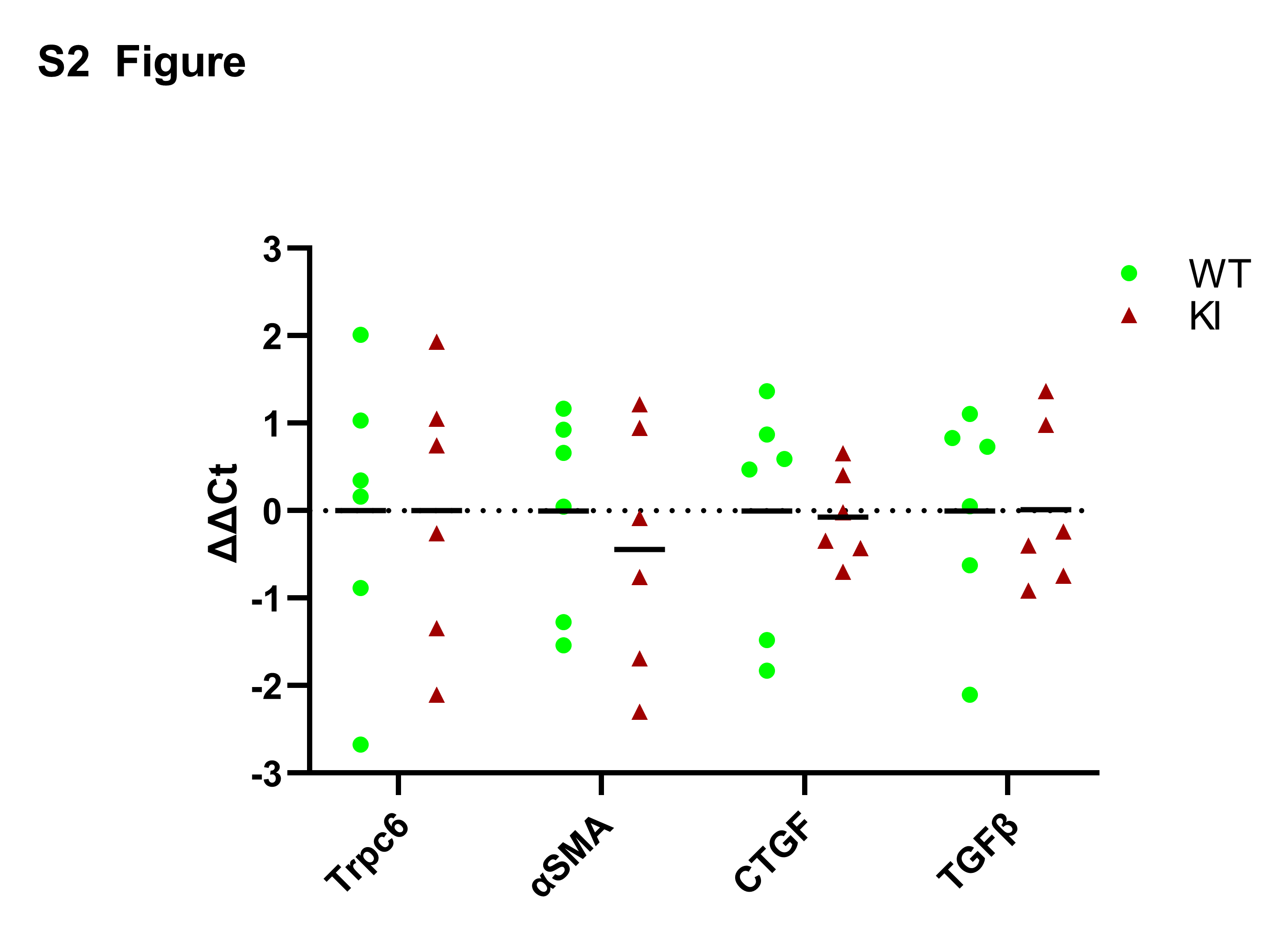

Supplement: S2 Fig — The relative mRNA expression levels of several fibrosis and renal injury related genes, and Trpc6, was compared in WT and KI male folate-nephropathy kidney samples (n = 6/group). There were no statistically significant differences between genotypes for any of the genes; multiple unpaired t-tests. (TIF) [file pone.0272313.s002.tif]

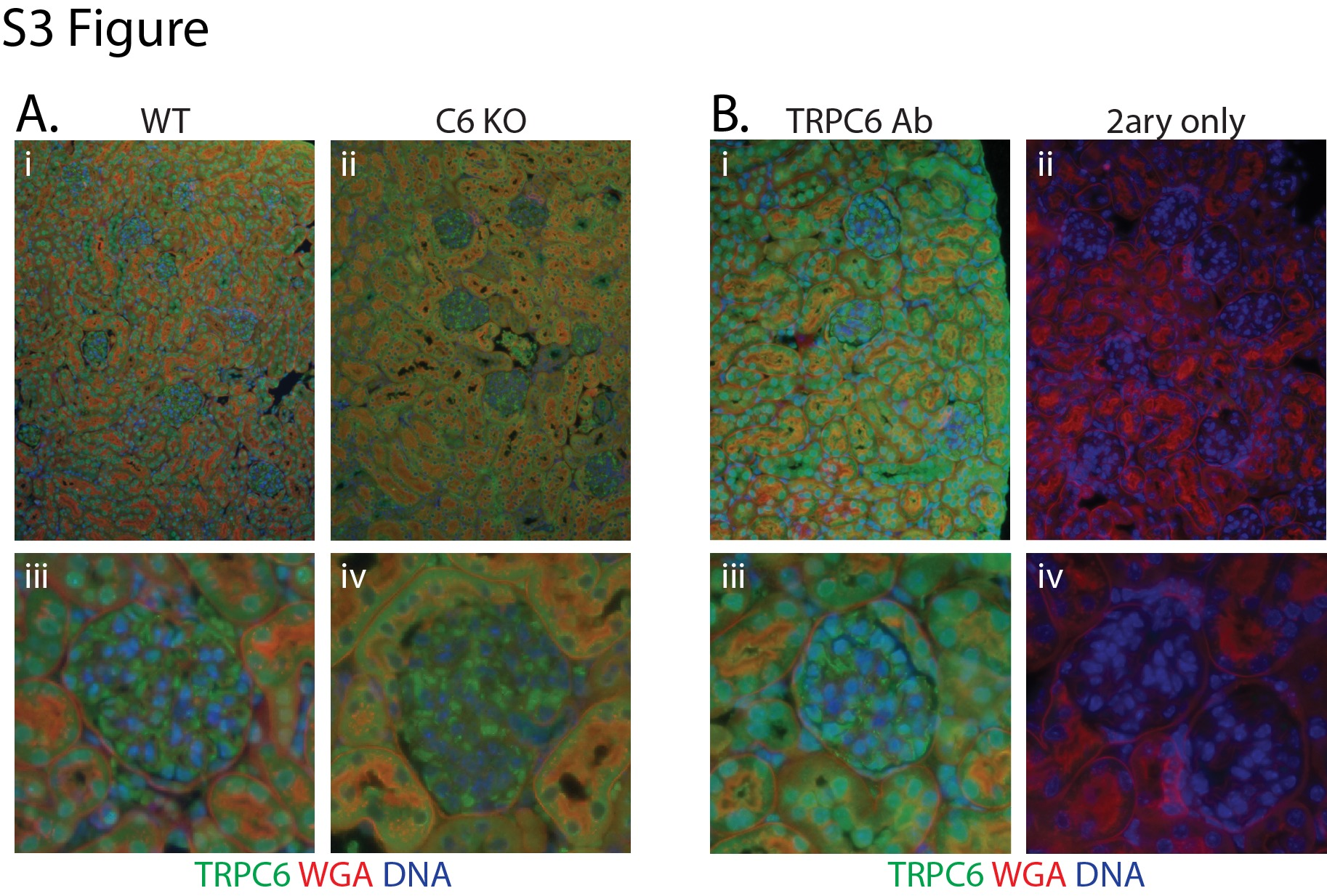

Supplement: S3 Fig — A, kidney sections from wild-type (i, iii), and Trpc6-/- (ii, iv) mice stained with rabbit anti-TRPC6 antibody (ACC-017, Alomone). Wheat germ agglutinin (WGA), and Hoechst were used as counterstains. TRPC6 staining specific to the wild-type kidney could not be identified. B, wild-type kidney sections were stained with TRPC6 antibody (i, iii) or with anti-rabbit secondary antibody only (ii, iv). Green channel signal was not due to non-specific secondary antibody staining or tissue auto-fluorescence. (TIF) [file pone.0272313.s003.tif]

Figure 6A TRPC6 Blot

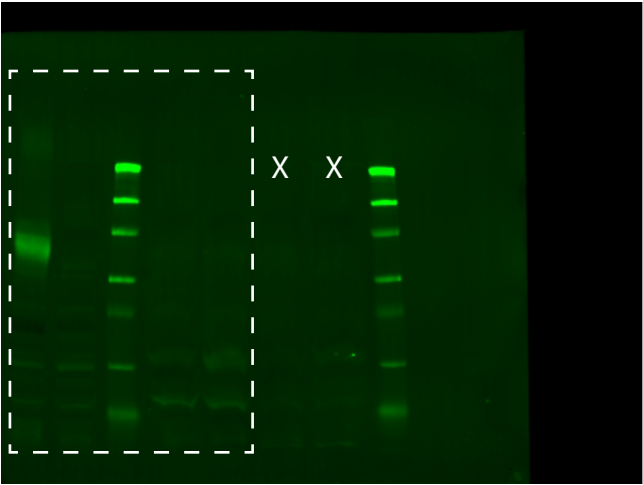

Figure 6A Erk1/2 Blot

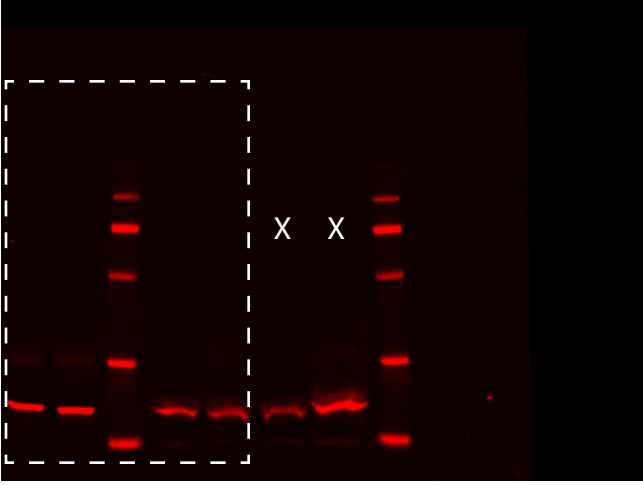

Figure 6A Podxl Blot

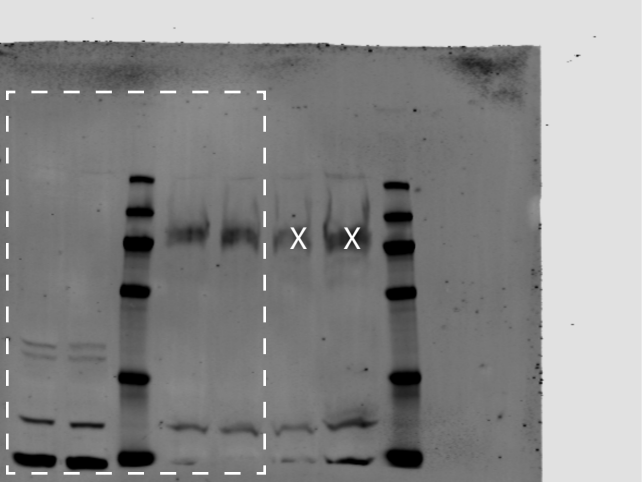

Supplement: S1 Raw images — (PDF) [file pone.0272313.s004.pdf]
